# Performance and pacing of professional IRONMAN triathletes: the fastest IRONMAN World Championship ever—IRONMAN Hawaii 2022

**DOI:** 10.1038/s41598-023-42800-z

**Published:** 2023-09-21

**Authors:** Beat Knechtle, Ivan Cuk, Elias Villiger, Pedro Forte, Mabliny Thuany, Marilia Santos Andrade, Pantelis T. Nikolaidis, Katja Weiss

**Affiliations:** 1 Medbase St. Gallen Am Vadianplatz, Vadianstrasse 26, 9001 St. Gallen, Switzerland; 2 Institute of Primary Care, University of Zurich, Zurich, Switzerland; 3 Faculty of Sport and Physical Education, University of Belgrade, Belgrade, Serbia; 4 Klinik für Allgemeine Innere Medizin, Kantonsspital St. Gallen, St. Gallen, Switzerland; 5 CI-ISCE, Higher Institute of Educational Sciences of the Douro, Penafiel, Portugal; 6 Department of Sports Sciences, Instituto Politécnico de Bragança, Bragança, Portugal; 7 Research Center in Sports, Health and Human Development, Covilhã, Portugal; 8 Faculty of Sport, University of Porto, Porto, Portugal; 9 Physiology Department, Federal University of Sao Paulo, São Paulo, Brazil; 10 School of Health and Caring Sciences, University of West Attica, Athens, Greece

**Keywords:** Environmental sciences, Environmental social sciences

## Abstract

Pacing during cycling and running in an IRONMAN triathlon has been investigated in only one study with elite IRONMAN triathletes. We have, however, no knowledge of how professional triathletes pace during an IRONMAN World Championship. To investigate the split-by-split speed, pacing strategies and pacing variability in professional female and male IRONMAN World Championship participants in the fastest IRONMAN World Championship ever in IRONMAN Hawaii 2022. For both cycling and running, 25 specific split times were recorded in each discipline. The best 30 men and 30 women overall were chosen from the official IRONMAN website database for further analysis. They were divided into three performance groups: Top 10, 11–20th place, and 21st–30th place. Mean speed, individual linear regressions with the corresponding correlation coefficients, and coefficient of variation were calculated to assess split-by-split speed, pacing strategies, and pacing variability, respectively. In both men’s and women’s cycling and running segments, the top ten participants exhibited faster split times compared to the slower performance groups. Notably, no discernible differences existed between the 11–20th and 21st–30th place in men’s cycling and women’s running times. Conversely, in men’s running and women’s cycling segments, those in the 11–20th place displayed quicker times than those in the 21st–30th place. In the cycling segment across all groups, men demonstrated a more negative pacing pattern (indicating an increase in speed), whereas women exhibited more consistent pacing. In the running segment, the top 10 men and all women’s groups showcased relatively similar slightly positive pacing profiles. However, men ranking 11–20th and 21st–30th displayed more pronounced positive pacing strategies, implying a more significant decline in speed over time. In terms of cycling, the variability in pacing remained relatively consistent across the three performance groups. Conversely, during the running segment, the top ten male triathletes and those in the 11–20th place displayed lower pacing variability than their counterparts in the 21st–30th position place and all women’s groups. In summary, performance and pacing were examined in professional male and female IRONMAN World Championship participants during IRONMAN Hawaii 2022. Top performers showed faster cycling and running split times, with differences in pacing strategies between sexes. The pacing was more consistent in cycling, while running pacing varied more, particularly among male triathletes in different performance groups.

## Introduction

The IRONMAN Hawaii has a long tradition and has been held for decades as the IRONMAN World Championship^1^. Over the years, both professional^2^ and age group^3^ IRONMAN athletes have improved their performance. In 2022, the fastest IRONMAN Hawaii ever was held with 10 male athletes breaking the 8-h barrier and the Norwegian Gustav Iden setting a new course record of 7:40:24 h:min:s, including a new running course record of 2:36:15 h:min:s. Furthermore, six women finished below the 9-h mark^4^. It is, however, already under discussion if athletes might be able to break the 7-h barrier^5^.

Pacing is an important aspect of endurance performance^6^ and has also been investigated in different triathlon distances^7^, including the IRONMAN distance^8–10^. In the IRONMAN triathlon, pacing is—among other variables such as age, previous experience, sex, training, origin, anthropometry, physiology, and performance in split disciplines—an important race predictor^11^. Pacing is the ability to appropriately distribute energy during a sports event and is typically modulated based on speed and intensity during the race to achieve optimal performance^12^. There are several studies about pacing in cycling^13,14^, swimming^15–17^ and running^18,19^. However, the literature still requires more studies of triathlon-based competitions, especially the IRONMAN. That said, it is interesting to examine the relative performance in the separate disciplines (swimming, cycling, and running) of triathlon, *i.e.*, whether a triathlete puts more effort in one discipline than in another, as well as within each discipline, especially in cycling and running where it is possible to study the variation of performance from one lap to another.

Considering the performance of Gustav Iden in IRONMAN Hawaii 2022 with both a new overall course record and a new course record in running, the question arises of which split discipline is the most important in an IRONMAN triathlon. A study investigating 343,345 athletes competing in 253 IRONMAN races found that the fastest IRONMAN triathletes were the relatively fastest in running and transition times^15^. Studies investigating elite IRONMAN triathletes reported different conclusions about the most predictive split. One of them observed that cycling was the most predictive split discipline^20^, another concluded that cycling and running were the most predictive^21^ while a different one showed that running is the most predictive^21,22^. Obviously, no consensus exists on which split discipline is the most predictive in an IRONMAN triathlon.

Independent of the question of which split discipline is the most predictive in an IRONMAN triathlon, only one study investigated the pacing in elite IRONMAN triathletes in cycling and running^23^. When analyzing 7687 cycling and 11,894 running split times of 1,392 IRONMAN triathletes, including 1263 men and 129 women, a positive pacing (*i.e.*, a decrease in speed) was reported for both women and men^23^. It is important to know that elite triathletes are no specific group since IRONMAN triathletes are separated into professional triathletes and others (*i.e.*, age group athletes or master athletes, or recreational athletes).

To date, we have no knowledge of how professional IRONMAN triathletes’ pace during an IRONMAN World Championship, especially not when several athletes broke the course record as it happened in 2022 in IRONMAN Hawaii. Knowledge about the way triathletes distribute their effort optimally among swimming, cycling, and running, as well as within disciplines, would be helpful in developing successful pacing strategies. Therefore, the aim of the study to investigate the split-by-split speed, pacing strategies and pacing variability in professional female and male IRONMAN World Championship participants in the fastest IRONMAN World Championship ever in IRONMAN Hawaii 2022. We compared pacing between the top 10, the 11th–20th place, and the 21st–30th place for both women and men for running and cycling. No comparison was possible for pacing in swimming because no electronically measured split times were available. Based upon existing data, we hypothesized (*i*) that running would be more predictive than cycling and (*ii*) pacing would be positive in both cycling and running for all performance groups independent of the sex of the athlete.

## Method

### Data set and data preparation

The race data were manually downloaded from the official IRONMAN website (www.ironman.com) where we selected the race data from the 2022 edition of ‘IRONMAN Hawaii’. The website recorded the name, sex, performance level (professional or age group athlete), country of origin, times of the split disciplines (swimming, cycling, and running), including the transition times (swimming to cycling and cycling to running). For both cycling and running, 25 specific split times were recorded. From the database, only the best 30 men and women were chosen for further analysis. They were consequently divided into three groups: Top 10, 11–20th place, and 21st–30th place.

### Statistical analysis

Prior to all analyses, descriptive statistics were calculated as mean, standard deviation, minimum and maximum. The Kolmogorov–Smirnov test and visual inspection of histograms and QQ plots confirmed the data distribution normality. To assess the split-by-split speed for cycling and running, two-way between-within ANOVAs were calculated separately for men and women. The main effects of *split speed* (25 splits), *ranking* (Top 10, 11–20th place, and 21st–30th place), and their interaction *split speed* × *ranking* was calculated.

Individual linear regressions were applied to the mean split speed for each of the 25 cycling and running laps. Individual linear regressions were calculated with the corresponding correlation coefficients (r) to assess pacing strategies (i.e., r > − 0.1 < 0.1 = Even pacing profile; r < − 0.1 = Positive pacing profile; r > 0.1 = Negative pacing profile). Furthermore, two-way between-between ANOVAs were performed separately on the Fisher Z-transformed correlation coefficients^24^ for cycling and running. The main effects of *sex* (men and women), *ranking* (Top 10, 11–20th place, and 21st–30th place), and their interaction *sex* × *ranking* were calculated, as well as Bonferroni post hoc test. The same two-way between-between ANOVAs were performed on the coefficient of variation (CV) to assess pacing variability. Since the CV data were expressed as percentages, data were log-transformed for the ANOVAs, and then back-transformed according to existing methods^25^. Eta squared (ŋ^2^) was calculated for the ANOVAs where the effect size 0.01, 0.06, and above 0.14 were considered small, medium, and large, respectively^26^. All correlation coefficients were interpreted as small, r = 0.10–0.29; moderate, r = 0.30–0.49; and large, r = 0.50–1.0 (Cohen, 2013). The level of statistical significance was set at *p* < 0.05. All statistical tests were performed using Microsoft Office Excel 2017 (Microsoft Corporation, Redmond, WA, USA) and SPSS 26 (IBM, Armonk, NY, USA).

### Ethics approval

This study was approved by the Institutional Review Board of Kanton St. Gallen, Switzerland, with a waiver of the requirement for informed consent of the participants as the study involved the analysis of publicly available data (EKSG 01/06/2010). The study was conducted in accordance with recognized ethical standards according to the Declaration of Helsinki adopted in 1964 and revised in 2013.

## Results

### Split times and speed

Descriptive data (*i.e.*, mean, standard deviation, minimum and maximum), specifically the time needed to complete each discipline, is shown in Table 1.

**Table 1:** Descriptive data of the time needed to complete each IRONMAN triathlon discipline.

	Men			Women		
	Top 10	11–20th place	21st–30th place	Top 10	11–20th place	21st–30th place
Swimming						
** Mean**	**0:50:08.80**	**0:49:47.30**	**0:49:48.40**	**0:55:22.10**	**1:00:13.40**	**1:05:47.70**
St. dev	0:02:00.96	0:01:41.07	0:01:26.69	0:02:36.55	0:04:40.36	0:07:34.78
Min	0:48:15.00	0:48:15.00	0:48:18.00	0:50:56.99	0:51:41.00	0:57:54.99
Max	0:52:57.00	0:52:50.00	0:51:44.00	0:58:08.00	1:04:56.00	1:19:47.00
Cycling						
** Mean**	**4:11:51.70**	**4:19:47.40**	**4:20:43.80**	**4:44:06.80**	**4:59:19.40**	**5:10:32.30**
St. dev	0:04:31.09	0:05:33.69	0:05:05.83	0:04:47.45	0:05:56.85	0:12:58.72
Min	4:04:35.99	4:09:02.99	4:14:16.00	4:36:10.00	4:51:56.00	4:57:09.00
Max	4:21:52.00	4:25:07.00	4:27:49.99	4:55:03.00	5:09:59.00	5:36:31.00
Running						
** Mean**	**2:44:01.50**	**2:51:31.10**	**3:04:59.30**	**3:09:06.50**	**3:17:02.90**	**3:26:37.70**
St. dev	0:04:25.76	0:05:06.05	0:04:45.75	0:10:29.76	0:08:05.55	0:14:05.71
Min	2:36:15.00	2:45:34.00	2:59:08.00	2:51:44.00	3:00:31.00	3:09:25.00
Max	2:49:28.00	3:02:16.00	3:13:31.00	3:23:44.99	3:26:30.00	3:49:20.00
Total						
** Mean**	**7:50:50.30**	**8:06:03.10**	**8:20:45.00**	**8:53:36.40**	**9:21:57.80**	**9:48:36.50**
St. dev	0:07:08.70	0:03:55.67	0:05:02.42	0:11:18.54	0:06:15.44	0:13:28.74
Min	7:40:24.00	8:00:50.00	8:13:40.00	8:33:46.00	9:11:03.00	9:32:56.99
Max	7:58:20.00	8:12:46.00	8:27:14.00	9:07:49.00	9:31:24.00	10:14:48.0

*Note that the transition times were outside our research focus, and consecutively, they are not included in this table. However, the total time consists of the transition times since the placement was based on that result.

The data were presented separately for sex and ranking. The importance of this specific race was in the fact that the first ten men completed the IRONMAN World Championship triathlon in under 8 h. Furthermore, 6 out of 10 women achieved a time of under 9 h. That makes this race by far the fastest IRONMAN World Championship ever. A split-by-split analysis was performed separately for men and women to investigate this race further.

**Figure 1:**
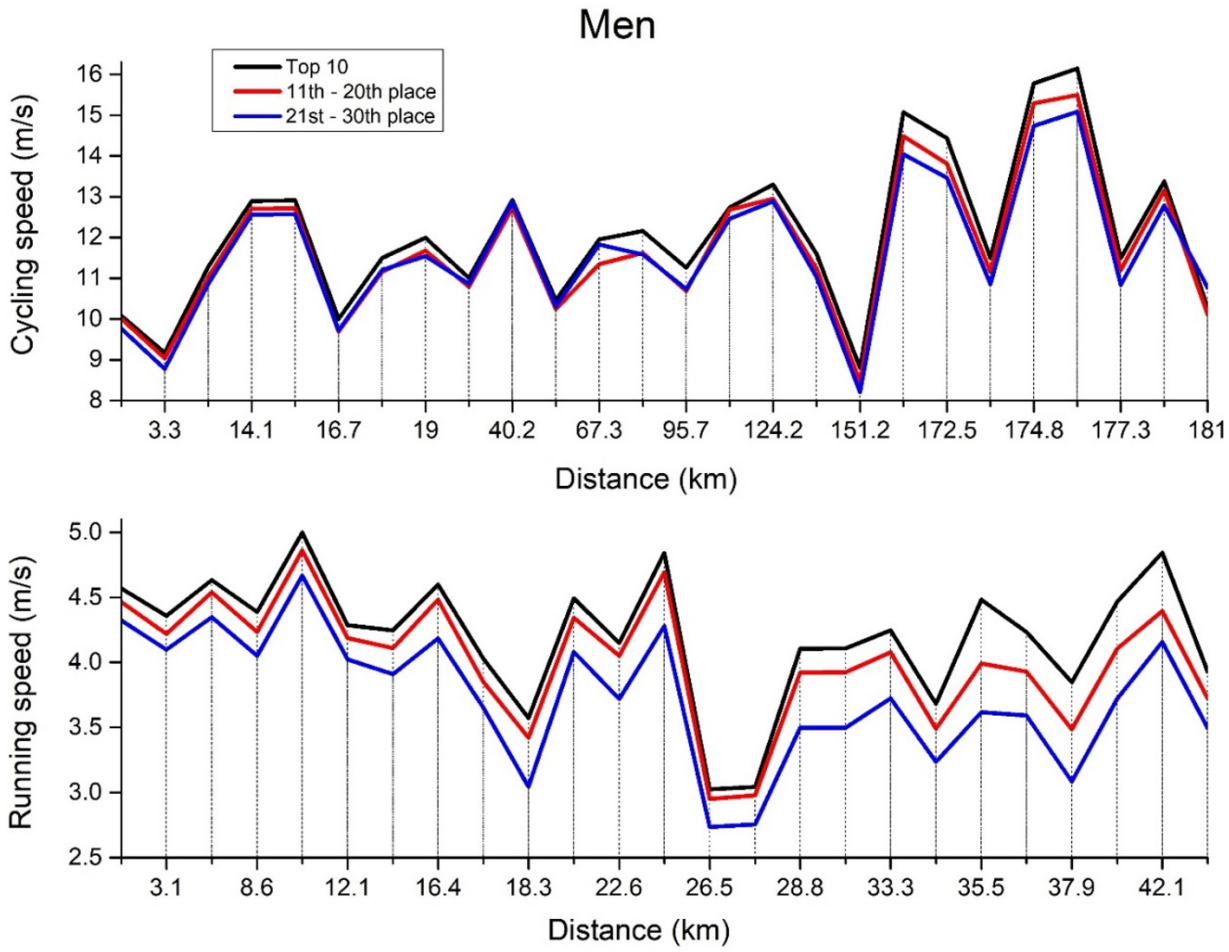
Men’s split-by-split cycling and running speed.

**Figure 2:**
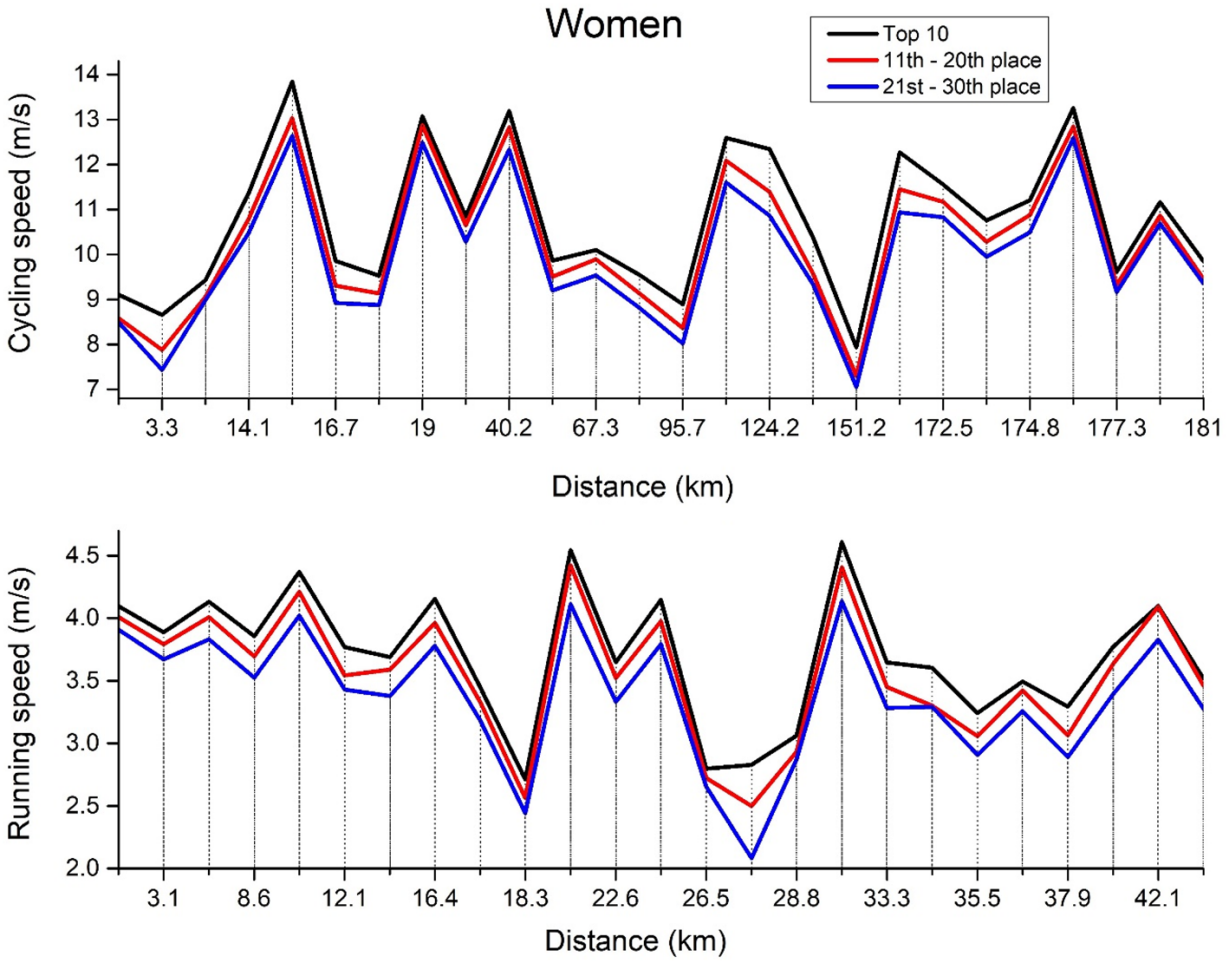
Women’s split-by-split cycling and running speed.

The two-way between-within ANOVAs were applied to assess split-by-split speed for cycling and running separately for men (Fig. 1) and women (Fig. 2). Regarding men cycling (Fig. 1, top panel), the significant main effects were observed in *split speed* [F_(2,24)_ = 331.4, ŋ^2^ = 0.896, *p* < 0.01] and *ranking* [F_(2,24)_ = 18.7, ŋ^2^ = 0.01, *p* < 0.01] but not in their interaction *split speed* × *ranking* [F_(2,24)_ = 1.31, ŋ^2^ < 0.01, *p* = 0.262]. In particular, each split speed differs from the other (*p* < 0.01), while the top 10 triathletes were significantly faster than the other two groups (*p* < 0.01). No significant differences were observed between the 11–20th and the 21st–30th place. In men running (Fig. 1, bottom panel), the significant main effects were observed in *split speed* [F_(2,24)_ = 322.6, ŋ^2^ = 0.761, *p* < 0.01], *ranking* [F_(2,24)_ = 55.4, ŋ^2^ = 0.123, *p* < 0.01] and in their interaction *split speed* × *ranking* [F_(2,24)_ = 4.08, ŋ^2^ = 0.02, *p* < 0.01]. In particular, each split speed differs from the other (*p* < 0.01) as well as each group (*p* < 0.01).

Regarding women cycling (Fig. 2, top panel), the significant main effects were observed in *split speed* [F_(2,24)_ = 697.5, ŋ^2^ = 0.899, *p* < 0.01], *ranking* [F_(2,24)_ = 27.8, ŋ^2^ = 0.04, *p* < 0.01] and in their interaction *split speed* × *ranking* [F_(2,24)_ = 2.13, ŋ^2^ < 0.01, *p* = 0.02]. In particular, each split speed differs from the other (*p* < 0.01) as well as each group (*p* < 0.01). In women running (Fig. 2, bottom panel), the significant main effects were observed in *split speed* [F_(2,24)_ = 208.6, ŋ^2^ = 0.748, *p* < 0.01] and *ranking* [F_(2,24)_ = 7.12, ŋ^2^ = 0.05, *p* < 0.01] but not in their interaction *split speed* × *ranking* [F_(2,24)_ = 1.11, ŋ^2^ < 0.01, *p* = 0.360]. In particular, each split speed differs from the other (*p* < 0.01), while only the Top 10 triathletes were significantly faster than triathletes from the 21st–30th place (*p* < 0.01).

### Pacing strategy

**Figure 3:**
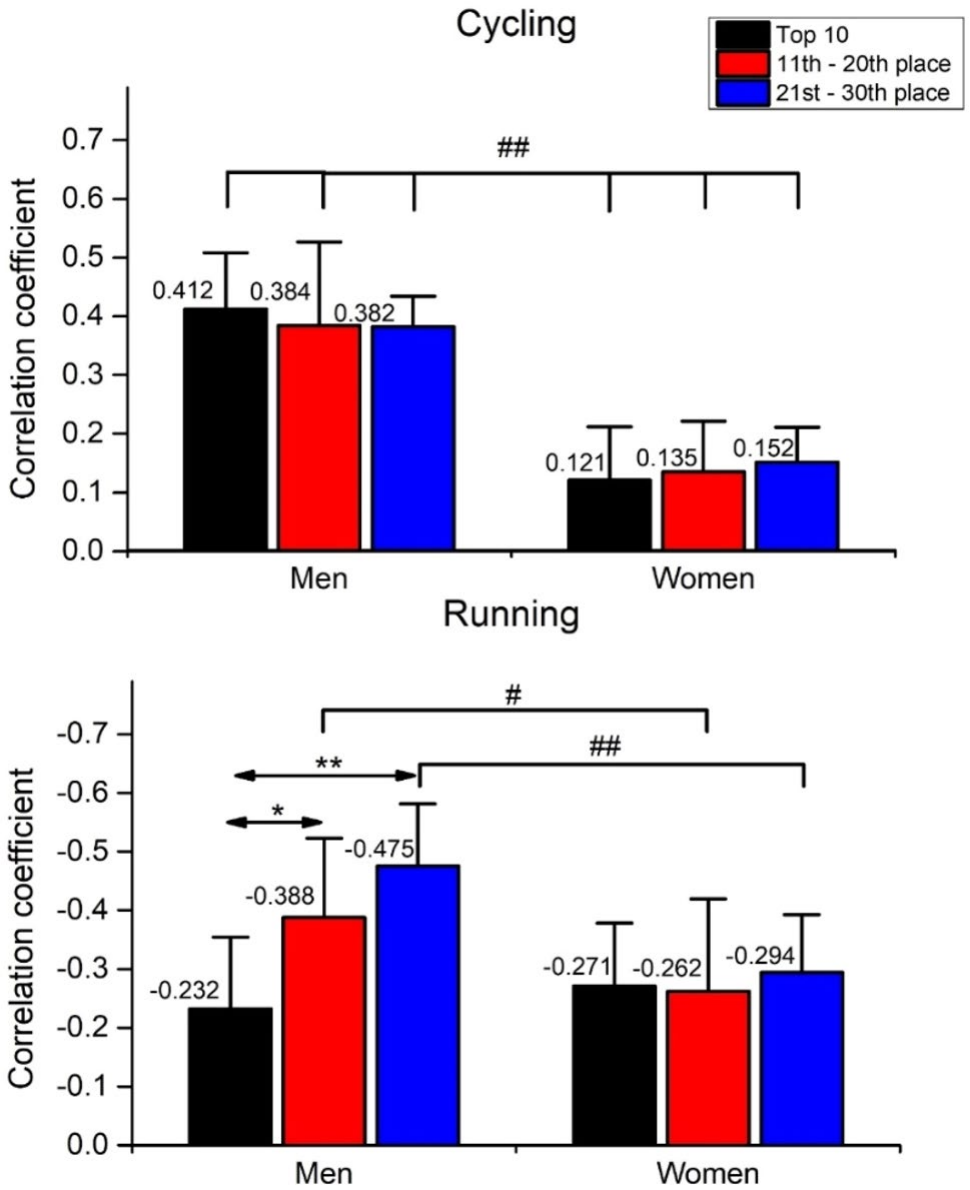
Mean correlation coefficients calculated from the individual linear regressions of the 25 cycling and running laps speed. r > − 0.1 < 0.1 = Even pacing profile; r < − 0.1 = Positive pacing profile; r > 0.1 = Negative pacing profile). #*p* < 0.05, ##*p* < 0.01 for main effect of *sex*. *p < 0.05, **p < 0.01 for the main effect of *ranking*.

To assess the triathletes` pacing strategy, the two-way between-between ANOVAs were performed on Fisher Z-transformed correlation coefficients (derived from individual linear regression) separately for cycling and running. In cycling (Fig. 3, top panel), the significant main effect of *sex* [F_(5,54)_ = 117.6, ŋ^2^ = 0.685, *p* < 0.01] was observed, while no significant effects were observed in the *ranking* [F_(5,54)_ = 0.03, ŋ^2^ < 0.01, *p* = 0.973] and *sex* × *ranking* interaction [F_(5,54)_ = 0.61, ŋ^2^ = 0.02, *p* = 0.548]. The Bonferroni post hoc test showed a significantly greater coefficient of correlation in men than in women in all groups (*p* < 0.01), indicating men’s more negative pacing profiles.

In running (Fig. 3, bottom panel), the significant main effects of *sex* [F_(5,54)_ = 117.6, ŋ^2^ = 0.685, *p* < 0.01], *ranking* [F_(5,54)_ = 0.03, ŋ^2^ < 0.01, *p* = 0.973] and the *sex* × *ranking* interaction [F_(5,54)_ = 0.61, ŋ^2^ = 0.02, *p* = 0.548] were observed. The Bonferroni post hoc test showed a significantly greater coefficient of correlation in men in the 11–20th (*p* < 0.05) and 21st–30th (*p* < 0.01) places than in the same women’s groups, indicating men’s more positive pacing profiles. Furthermore, men`s top 10 triathletes showed a significantly lower coefficient of correlation than triathletes from the 11–20th (*p* < 0.05) and 21st–30th (*p* < 0.01) place, indicating less positive pacing strategy.

### Pacing variability

**Figure 4:**
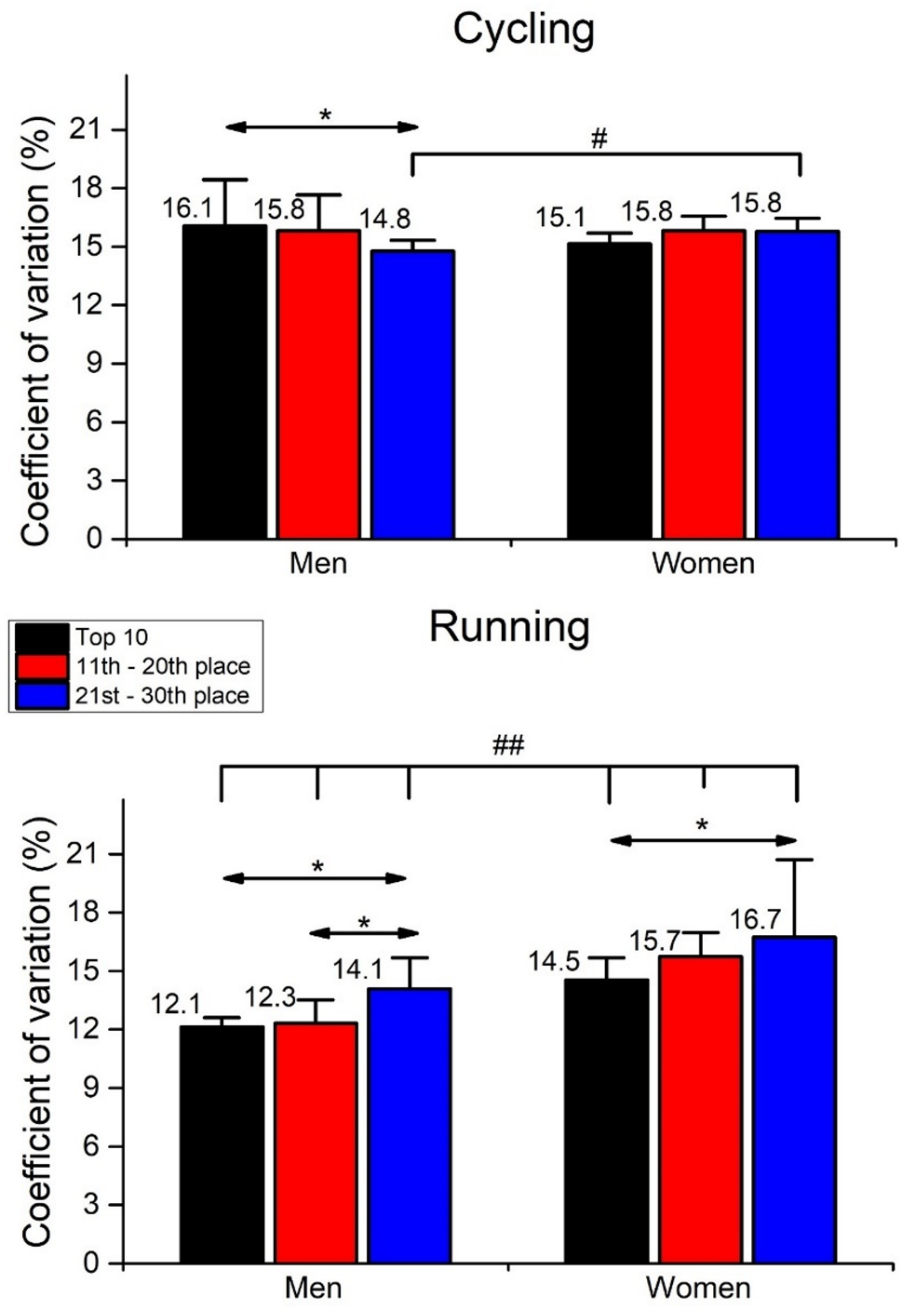
Mean coefficient of variation (%) of the 25 cycling and running laps speed. #*p* < 0.05, ##*p* < 0.01 for main effect of *sex*. *p < 0.05, for the main effect of *ranking*.

To assess the triathletes` variability in pacing, the two-way between-between ANOVAs were performed on the coefficient of variation (CV). Since the CV data were expressed as percentages, data were log-transformed for the ANOVAs, and then back-transformed. In cycling (Fig. 4, top panel), no significant main effect of *sex* [F_(5,54)_ = 0.10, ŋ^2^ < 0.01, *p* = 0.754] and *ranking* [F_(5,54)_ = 0.95, ŋ^2^ = 0.03, *p* = 0.392] were observed, while *sex* × *ranking* interaction was significant [F_(5,54)_ = 3.917, ŋ^2^ = 0.06, *p* = 0.043]. The post hoc analysis showed that men triathletes placed from the 21st–30th place have lower pacing variability than the top 10 men and women placed from the 21st–30th place (*p* < 0.05).

Contrary to cycling, in running (Fig. 4, bottom panel), the significant main effect of *sex* [F_(5,54)_ = 48.5, ŋ^2^ = 0.474, *p* < 0.01] and *ranking* [F_(5,54)_ = 7.98, ŋ^2^ = 0.228, *p* < 0.01] were observed, while their interaction *sex* × *ranking* interaction was not significant [F_(5,54)_ = 0.92, ŋ^2^ = 0.03, *p* = 0.405]. The Bonferroni post hoc test showed that all women’s groups have more running variability than men`s (*p* < 0.01). Furthermore, men triathletes from the 21st–30th place had more variability in the running than the other two groups (*p* < 0.05). In comparison, women triathletes from the 21st–30th place had more variability than the top 10 group (*p* < 0.05).

## Discussion

This study intended to investigate the split-by-split speed, pacing strategies, and pacing variability in professional female and male IRONMAN World Championship participants in the fastest IRONMAN World Championship ever in IRONMAN Hawaii 2022. By comparing pacing between the top 10, the 11–20th place, and the 21st–30th place for both sexes, we hypothesized for all performance groups that running would be more predictive of overall performance than cycling, and pacing would be positive (*i.e.*, the speed would decrease across the split) in both cycling and running.

The main findings were as follows:

1. The top 10 were faster in cycling and running than lower-placed athletes

The first important finding was that ten fastest women and men were faster than athletes in both cycling and running in the 11–20th and 21st–30th place. In contrast, the top 10 male IRONMAN triathletes had slower swimming times (Table 1) than other groups. However, no split times were provided for swimming, thus not allowing further investigation. For the two slower performance groups, the 11–20th were faster than the 21st–30th place in the men’s running and women’s cycling, implying that men would need to improve running and women cycling to enter the top twenty in IRONMAN Hawaii, and athletes who were in the 11–20th group would need to improve both running and cycling to enter to top ten in IRONMAN Hawaii.

The first important finding was that the top ten male and female athletes surpassed those in 11–30th positions in both cycling and running. In contrast, the leading male IRONMAN triathletes showed slower swimming times (Table 1) compared to other groups, with no available split times for further analysis. Among the slower performance groups, those ranked 11–20th showed better performance in men’s running and women’s cycling than those in 21st–30th positions. This highlights the necessity for men to enhance their running and women to improve their cycling to secure a top twenty position in IRONMAN Hawaii. Moreover, athletes in the 11–20th group would need to excel in both running and cycling to attain a top ten position in the competition.

Little was known regarding pacing in the IRONMAN triathlon^23^. More studies investigated pacing in shorter^27^ or longer^28^ triathlon distances. Especially, little to nothing was known regarding pacing in triathlons by performance groups. In the Sprint distance, Olympic distance, and the IRONMAN 70.3 distance triathlon races, an even swimming pacing strategy was adopted across all distances. During cycling, pacing varied for the three distances. In running, the pacing was negative in the Sprint distance triathlon and positive in the Olympic distance and the IRONMAN 70.3 distance triathlon^27^. In races covering x-times the full IRONMAN distance, the fastest triathletes spent relatively more time swimming and cycling and less time running, showing the importance of running in longer triathlon races^28^. Future studies would need to investigate differences between performance groups in professional IRONMAN triathletes regarding anthropometry, training, and equipment. This might provide further insights in the performance differences of the fastest professional IRONMAN triathletes.

2. Differences in pacing strategies during cycling and running between women and men

A second important finding was that men overall paced more negatively in cycling while women paced rather evenly, whereas in running the ten fastest men and all three female groups paced slightly more positive. Men in the two slower performance groups showed more pronounced positive pacing implying that slower male triathletes might try to adopt more even pacing to improve results.

The differences between the sexes might be explained by differences in anthropometry and training in female and male triathletes^29–31^. Female triathletes had a lower body mass, a lower body height, a lower skeletal mass and a lower BMI compared to male triathletes^30^, but a higher percent body fat^30,31^. It is reported that female triathletes invested fewer weekly training hours on average and covered fewer training kilometers in all split disciplines than male triathletes^31^. Furthermore, swimming and cycling speed in training was higher in male triathletes^30^. However, running speed in training was higher in female triathletes^30^. Sex differences between the energetic metabolisms could also be associated with the fact that the women paced rather evenly. Women showed a greater proportional area of type I muscle fibers, economize more carbohydrates during prolonged exercise, and are more effective in the usage of fatty acids than the males, demonstrating a more uniform pacing strategy and less fatigue following long-distance aerobic exercise^32^. It has also been shown that variables of anthropometry and training were differently related to overall race times in female and male triathletes^31^. For example, it has been reported that the body fat percentage is related to the overall race times in male triathletes^31^, where average weekly training volume was not related^30^. In contrast, in female triathletes, the average weekly training volume was related to the overall race times^30,31^. Another study found that the somatotype was a strong predictor in male triathletes. For female triathletes, however, no correlation between somatotype, training, and the race could be found^29^. This highlights the lack of research on female triathletes, where anthropometrics, training intensity and volume, and racing performance seem to be important topics for future research.

Psychological differences between males and females have an effect on pacing as a result of competitiveness. Males are widely recognized for their stronger inclination toward direct competition in contrast to females^33^, a characteristic often attributed to their heightened sense of competitiveness^34^. This affects the pacing strategy in the form of a more aggressive start resulting in a steeper decline.

3. Differences in pacing variability between performance groups and sexes

A third and last important finding was that pacing variability did not change across the three performance groups in cycling. It should be highlighted that the athletes adopted a negative pacing strategy (*i.e.*, an increase in speed during the race) in cycling. An interpretation of this pacing strategy from a biophysical perspective might be the elevations of the course, especially considering the decrease of elevation towards the end of the cycling race. It has been proposed that an optimal pacing strategy is increasing power in uphill sections and decreasing power when traveling downhill^35,36^.

Drafting during running also has an effect on the running performance^37^ and is permitted in an IRONMAN triathlon. However, drafting is less effective in running than in cycling due to the lower achieved speed^38^. As a result, we might expect that less prepared triathletes will vary more in marathon pacing due to a slowdown caused by an inability to stay in the pack. Furthermore, frequent acceleration and deceleration can increase the risk of injury due to greater impact forces on the musculoskeletal apparatus^39^.

Particularly novel findings of this study were that the two fastest male performance groups showed a lower running variability (*i.e.*, a more even pacing) than the slowest performance group and all three female performance groups. However, previous studies showed that women paced more evenly in marathons than men^40,41^. In contrast, differences seemed to exist between the sexes regarding pacing in marathon running since another study reported that men seemed to slow more in a marathon than women^42^. Little is known, however, regarding the sex differences in pacing in triathlon. In ULTRAMAN Hawaii, a triathlon covering three stages during three days, women paced differently than men. Performance in the fastest women decreased on day 1 but was maintained on days 2 and 3. Performance in the fastest men decreased on day 1 and day 2 but improved on day 3^43^. Future studies need to investigate reasons for pacing differences between female and male triathletes.

**Figure 5:**
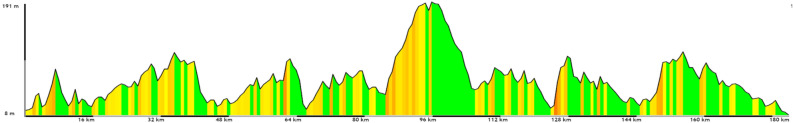
The course profile for the cycling split.

**Figure 6:**
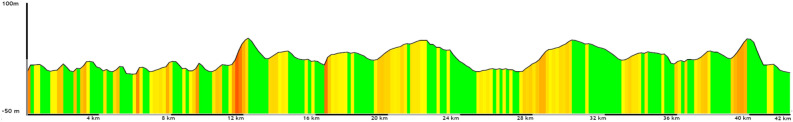
The course profile for the running split.

Evidently, the pacing variability seems not quite as important for professional triathletes in cycling, while less variable groups achieve better results in the running. In particular, slower men and all women might try reducing pacing variability to achieve better results. To date, very little is known regarding the sex differences in triathlon performance^44^ and in pacing during triathlon^45,46^. Stevenson et al. investigated the sex differences in performance in the top ten female and male age group triathletes for age groups 18–24 to 60–64 years for Sprint, Olympic, and IRONMAN 70.3 triathlons during the 2009–2011 World Championships. Sex differences in overall race times were highest in both the youngest and the oldest age groups for all triathlon distances, where the largest sex differences existed for swimming^44^. Le Meur et al.^45^ investigated the differences in pacing between female and male elite triathletes a World Cup Olympic distance competition with drafting during cycling. Speeds in swimming and running decreased similarly for both women and men. Male athletes were faster than women in the transition from swimming to cycling, pushing the pace harder during the swim-to-cycle transition, contrary to the women. Women showed more changes in speed due to sloping changes during cycling and running^45^. However, drafting is allowed in Olympic distance triathlon but not in the full IRONMAN distance triathlon; therefore, comparisons are difficult. A study investigating pacing in multi-stage triathlons covering the daily distance of 5-times, 10-times, and 20-times the full IRONMAN distance showed that men achieved a stable cycling performance whether they competed in 5-times, 10-times, or 20-times the full IRONMAN distance. The cycling performance influenced the subsequent running split depending upon whether they competed in 5-times, 10-times, or 20-times the full IRONMAN distance^46^. Future studies need to investigate the pacing differences between the sexes and the split disciplines more deeply. We also need to consider the course profile in cycling (Fig. 5) and running (Fig. 6), showing that the racecourse is not flat. Up-hill and down-hill sections might differently influence female and male cycling and running performance^8,10^.

### Limitations

Τhis study is not free of limitations since aspects of food^47^ and fluid^48^ intake, the influence of dehydration^49^ and heat^50^, mental toughness^51^ and equipment^51^ for IRONMAN could not be included. On the other hand, its strength is its novelty and practical applications considering that this data analysis provides insights on the pacing strategy of the fastest professional IRONMAN triathletes.

## Conclusion

In summary, this study investigated split-by-split speed, pacing strategies, and pacing variability in male and female IRONMAN World Championship participants during the IRONMAN Hawaii 2022 race. The analysis revealed that the top ten performers exhibited faster cycling and running split times compared to their slower counterparts, while differences in pacing strategies emerged between men and women, with men showing more negative pacing during cycling and women maintaining a steadier pace. Additionally, pacing variability remained consistent in cycling across performance groups, whereas in running, differences were observed with lower pacing variability seen in the top ten male triathletes and those ranking 11–20th compared to those in the 21st–30th position and all female groups. These findings highlight the significance of pacing strategies in IRONMAN triathlons and suggest areas for further research into factors influencing pacing differences and their impact on overall performance.

## Data Availability

All athlete data was downloaded from the official IRONMAN website (www.ironman.com).
